# The role of sociodemographics, behavioral factors, and internet use behaviors in students' psychological health amid COVID‐19 pandemic in Bangladesh

**DOI:** 10.1002/hsr2.398

**Published:** 2021-10-01

**Authors:** Ismail Hosen, Firoj al Mamun, Mohammed A. Mamun

**Affiliations:** ^1^ CHINTA Research Bangladesh Dhaka Bangladesh; ^2^ Department of Public Health and Informatics Jahangirnagar University Dhaka Bangladesh

**Keywords:** COVID‐19, psychological impact, depression, anxiety, internet use behaviors, Bangladeshi students

## Abstract

**Background:**

The COVID‐19 pandemic drastically impacted students' psychological well‐being by interchanging their regular activities. Students are more engaged with online activities, which may affect their mental health. Therefore, the present study aims to investigate the magnitude of psychological health of the Bangladeshi students, where the role of online use behaviors is also investigated.

**Methods:**

An online‐based cross‐sectional survey was conducted between 7 October 2020 and 2 November, 2020 among Bangladeshi students utilizing a convenience sampling technique. The survey questionnaire included items concerning sociodemographics, behavior and health‐related variables, online use behaviors, Patient Health Questionnaire (PHQ‐2), and Generalized Anxiety Disorder (GAD‐2). Descriptive and inferential statistics were used to present the data (i.e., binary logistic regression was performed to examine the association between variables and hierarchical regression analysis was performed to predict the variance for depression and anxiety).

**Results:**

Out of 601 included students, 260 and 196 students reported the symptoms of depression (43.3%) and anxiety (32.6%), respectively, where female students experienced a higher level of psychological problems. The psychological suffering was also significantly associated with medical college students, having an affair, less sleep time, not performing physical exercise, excessive internet use, and not watching news during online activities. After adjusting all of the studied variables in the hierarchical regression models, it explained 10% and 9.6% variance of depression and anxiety, respectively.

**Conclusions:**

During this pandemic situation, a relatively higher level of psychological complications was observed among the Bangladeshi students. Regrettably, any specific policy was absent in the country to assuage that outcome. Therefore, based on the study finding, a few strategies and policies were recommended that may be helpful to alleviate psychological issues among the students.

## 1 INTRODUCTION

The outbreak of novel coronavirus disease 2019 (COVID‐19) has disrupted the quality of life as well as the way of living across the world. Under unexpected circumstances, the authority of Bangladesh, like other countries, took a rapid response by implementing a countrywide lockdown in suppressing the rapid transmission of the virus.^1^ During the COVID‐19 outbreak like previous outbreaks (e.g., SARS in 2003, Ebola in 2014), preventive measures (e.g., restricted social movement, limited transport facilities, traveling confinement with other countries, quarantine or isolation for suspected or infected cases along with mandatorily maintaining personal hygiene practices such as frequent hand washing and wearing a mask) are imposed in various countries including Bangladesh to curb the outbreak.^2^, ^3^, ^4^ These measures are undoubtedly effective for reducing virus transmission. More specifically, a recent review assessing the effects of quarantine finds that 44% to 96% and 31% to 76%, incident and death rates, respectively, averted compared with no quarantine measures taken scenario.^5^ This confinement situation is also being alleged for elevating public mental suffering by aggregating common psychological stressors such as (a) longer duration of quarantine, (b) fear of being infected, (c) feeling of frustration and boredom, (d) inadequate basic supplies (e.g., food, water, clothes), (e) inadequate information and misinformation, (f) stigma, (g) financial constriction, and so on.^6^ These stressors are more common in Asian countries where most of the countries are developing; therefore, the impact of COVID‐19 pandemic adversely affected physical as well as mental well‐being during this pandemic situation including Bangladesh.^4^, ^7^ However, the stressors mentioned earlier are also leading to suicide in extreme psychological conditions^8^, ^9^ which is also supported by global findings.^10^, ^11^, ^12^

Although quarantine for the suspected cases is mandatorily imposed in almost all countries, some individuals do not feel its necessity. For instance, in the SARS outbreak period in 2003, 15% of the quarantined people do not think they should be placed into quarantine at all.^13^ The same study also reported higher mental health suffering (e.g., post‐traumatic stress disorder, depression, and anxiety) among the quarantined individuals in Toronto, Canada.^13^ In the present COVID‐19 context, all educational institutions were mandatorily closed down since the pandemic inception as a preventive approach for mitigating the virus transmission among the students.^8^, ^14^ Thus, typical schooling has been converted into online schooling, leading to changes in the students' social circle, communication, and interaction.^8^, ^15^ Online schooling can be uncomfortable to some students as issues like problems in understanding materials, limited access to online schooling materials, technical issues, disturbance in the flow of classes, and lack of interest or motivations to attend classes.^8^, ^16^, ^17^ For instance, a Bangladeshi mother–son suicide pact is reported because of parental conflict raised by online schooling.^18^ In general, the pandemic‐related consequences are as follows, (a) loneliness as of interrupting social interaction, (b) social and economic disruption, (c) fear of losing beloved one, (d) uncertainty about their future regarding their education and career are common among the students, which later on develops mental disruption.^8^, ^19^, ^20^ A few epidemiological studies were directed among the Bangladeshi students, mainly university,^21^, ^22^, ^23^, ^24^ and medical college students.^25^ But yet, no studies directed including various categories of students herein.^8^ Recently a systematic review reported higher mental health suffering among the Bangladeshi students, where depression and anxiety were reported up to 80% and 90%, respectively,^8^ which was remarkably higher than the general people.^26^

Students are heavily engaged with technology and the role of internet during this pandemic to spend leisure time and/or attend online schooling. Prior to the pandemic, a study conducted in an Asian country indicated that young people were involved in using internet for the purpose of playing online games mostly, which lead them into addictive behaviors toward internet.^27^ Thus, excessive technology use leads to addictive behaviors, which are also linked with poor mental health.^28^, ^29^ In extreme cases, technology‐related addictive behaviors may lead to self‐harm attitudes. For instance, excessive online use and gaming‐related suicides are reported in the non‐COVID‐19 and COVID‐19 periods.^30^, ^31^ Nevertheless, there is a shortage of studies assessing youth's mental health concerning their online engagement during the COVID‐19 pandemic in Bangladesh.^8^ More specifically, the role of exposure in social and electronic media in the students' generalized anxiety disorder (GAD) is investigated by a study.^32^ But the study failed to observe any relationships between internet use behaviors and psychological problems, which is investigated herein. Besides, the present study also presented the predictive models for students' mental health problems for the first time in this context.

## 2 METHODS

### 2.1 Study procedure, participants, and ethics

A cross‐sectional online study was carried out among the Bangladeshi students. Data were collected between 7 October and 2 November, 2020 through an online‐based data collection tool (e.g., Google Form). Participants were recruited from the entire country utilizing a convenience sampling technique. A structured questionnaire was circulated across different social media sites to collect data (i.e., Facebook, WhatsApp). The study participation was voluntary, where online informed consent has been taken from the respondents after exploring the aims and purpose of the study. Besides, the confidentiality and anonymity of their data were also ensured. Initially, 617 respondents completed the survey, but after eliminating the incomplete survey, a total of 601 data were considered for final analysis in the study. To participate in the survey, inclusion criteria were being active Bangladeshi students (≥high school), having internet access, and their interest in the study. Prior study implementation, a formal ethical approval was obtained from the ethical committee of the Institute of Allergy and Clinical Immunology of Bangladesh, Savar, Dhaka, Bangladesh. Besides, other ethical aspects as provided by the Helsinki Declaration of 2013 were also adhered to the study.

### 2.2 Sample size determination

The sample size was calculated based on the following established formula. However, considering the below‐mentioned formula, the estimated sample size was 385.

$$\text{Sample size}=\frac{\frac{z^{2}p\left(1-p\right)}{e^{2}}}{1+\frac{z^{2}p\left(1-p\right)}{e^{2}N}},$$

where *N* is the population size, infinite; *e* is the margin of error, 0.05; *z* is the *z*‐score, 1.96 (95% confidence level); *p* is the prevalence rate estimation, 50%.

### 2.3 Measures

#### 2.3.1 Sociodemographic factors

Basic sociodemographic information was collected concerning gender, educational status (e.g., university, medical college, high school), residence area (e.g., rural or urban), relationship status (i.e., single, in a relationship, and married), monthly family income (e.g., middle class = 15,000–30,000 BDT), family types (e.g., joint or nuclear, where a nuclear family consists only of parents and their children), and whether they are living with their family or not at this survey time.

#### 2.3.2 Behavioral health‐related measures

Behavioral health‐related information was collected concerning sleeping status (e.g., normal sleeping status was considered 6–7 hours), physical exercise (e.g., at least 30 minutes walking, cycling, swimming, or other activities regularly), smoking status, and perceived health status (e.g., asthma, heart problems, kidney problems, diabetes).

#### 2.3.3 Internet use behaviors

Internet use‐related information was also assessed in the study, where the duration of internet use was assessed utilizing a previously deployed category in the Bangladeshi study.^28^ Following the earlier study, internet use purposes were also included, such as educational activities, messaging, gaming, watching videos, social media browsing, online shopping, watching the news, and others where students could choose multiple responses according to their application.

#### 2.3.4 Depression

Depression was assessed by using the Patient Health Questionnaire (PHQ‐2) scale.^33^, ^34^ Participants were asked how often they experienced the two core symptoms of depressive disorder over the past two weeks (i.e., “Little interest or pleasure in doing things”, and “Feeling down, depressed, or hopeless”), and their responses were recorded on a 4‐point Likert scale (0 = not at all, 1 = several days, 2 = more than half the days, 3 = nearly every day) score ranging from 0 to 6 where a total score of ≥3 was indicating the presence of depression.^33^, ^34^ The Cronbach's alpha was 0.76.

#### 2.3.5 Anxiety

Anxiety was assessed by using the Generalized Anxiety Disorder (GAD‐2) scale.^33^, ^35^ Participants were asked how often they experienced the two core symptoms of anxiety disorder over the past two weeks (i.e., “Feeling nervous, anxious, or on edge”, and “Not being able to stop or control worrying”), and their responses were recorded on a 4‐point Likert scale (0 = not at all, 1 = several days, 2 = more than half the days, 3 = nearly every day)^33^, ^35^ score ranging from 0 to 6 where a total score of ≥3 was indicating the presence of anxiety.^33^ The Cronbach's alpha was 0.76.

### 2.4 Statistical analysis

Data were analyzed by using IBM SPSS Statistics version 22 and Microsoft Excel 2019. At first, the collected data were cleaned and prepared for final analysis by Microsoft Excel 2019. The SPSS was used to perform descriptive statistics such as frequencies, percentages. Inferential statistics such as Chi‐sqaure, and binary logistic regression tests was used to identify associate factors related to depression and anxiety. Moreover, a hierarchical regression analysis was performed where sociodemographic, behavioral health, and internet use‐related variables were included as predictors of depression and anxiety. The normality of distribution and multicollinearity (e.g., VIF and tolerance values) were also tested. A *P‐*value <.05 was used as statistically significant.

## 3 RESULTS

### 3.1 Characteristics of the participants

Among the participants, more than half of the respondents were male (57.2%), 65.6% were university students, 75.2% from urban areas, and 44.6% from the upper‐class family. In contrast, most of them were from nuclear families, single in relationship status, and currently living with family. In addition, half of the respondents were involved in physical exercise, whereas 6 to 7 hours as sleeping time was reported in 53.9% of them, and 53.2% used the internet for more than 5 hours. In contrast, 91.5% and 89.2% of the students were not involved with smoking and perceived health status as bad, respectively (Tables 1, 2, and 3).

**TABLE 1:** Associations between the sociodemographic variables and mental health problems

Variables	Total sample (n, %)	Depression (260; 43.3%)				Anxiety (196; 32.6%)			
		Yes (n, %)	χ^2^ test value	*P*‐value	Odds ratio (95% CI)	Yes (n, %)	χ^2^ test value	*P*‐value	Odds ratio (95% CI)
**Gender**									
Male	344 (57.2)	139 (40.4)	2.670	.102	0.762 (0.550‐1.056)	101 (29.4)	3.871	.049	0.709 (0.503‐0.999)
Female	257 (42.8)	121 (47.1)			Reference	95 (37)			Reference
**Educational status**									
University	394 (65.6)	161 (40.9)	4.026	.134	1.131 (0.520‐2.458)	120 (30.5)	6.359	.042	1.679 (0.667‐4.228)
Medical college	178 (29.6)	88 (49.4)			1.600 (0.715‐3.581)	70 (39.3)			2.485 (0.963‐6.408)
High school	29 (4.8)	11 (37.9)			Reference	6 (20.7)			Reference
**Current residence**									
Rural	149 (24.8)	68 (45.6)	0.456	.500	1.137 (0.783‐1.650)	47 (31.5)	0.103	.748	0.937 (0.630‐1.394)
Urban	452 (75.2)	192 (42.5)			Reference	149 (33)			Reference
**Monthly family income (BDT)**									
<15000	106 (17.6)	47 (44.3)	0.240	.887	1.093 (0.694‐1.720)	33 (31.1)	0.520	.771	0.973 (0.599‐1.581)
15000‐3000	227 (37.8)	100 (44.1)			1.080 (0.756‐1.543)	78 (34.4)			1.127 (0.774‐1.641)
>30000	268 (44.6)	113 (42.2)			Reference	85 (31.7)			Reference
**Family type**									
Joint	132 (22.0)	55 (41.7)	0.175	.676	0.920 (0.622‐1.360)	44 (33.3)	0.040	.841	1.043 (0.692‐1.572)
Nuclear	469 (78.0)	205 (43.7)			Reference	152 (32.4)			Reference
**Relationship status**									
Single	478 (79.5)	208 (43.5)	12.562	.002	2.311 (1.229‐4.345)	150 (31.4)	8.803	.012	1.372 (0.727‐2.589)
In a relationship	67 (11.1)	38 (56.7)			3.931 (1.812‐8.526)	32 (47.8)			2.743 (1.268‐5.935)
Married	56 (9.3)	14 (25)			Reference	14 (25)			Reference
**Currently living with family**									
No	78 (13.0)	26 (33.3)	3.599	.058	0.618 (0.374‐1.019)	22 (28.2)	0.792	.373	0.788 (0.466‐1.333)
Yes	523 (87.0)	234 (44.7)			Reference	174 (33.3)			Reference

**TABLE 2:** Associations between behavioral health‐related variables and mental health problems

Variables	Total sample (n, %)	Depression (260; 43.3%)				Anxiety (196; 32.6%)			
		Yes (n, %)	χ^2^ test value	*P*‐value	Odds ratio (95% CI)	Yes (n, %)	χ^2^ test value	*P*‐value	Odds ratio (95% CI)
**Daily sleeping hour**									
Less than 6 hours	69 (11.5)	36 (52.2)	5.377	.068	1.248 (0.724‐2.154)	35 (50.7)	13.423	.001	2.029 (1.168‐3.527)
6 to 7 hours	324 (53.9)	127 (39.2)			0.738 (0.519‐1.049)	91 (28.1)			0.770 (0.529‐1.121)
More than 7 hours	208 (34.6)	97 (46.6)			Reference	70 (33.7)			Reference
**Physical exercise**									
No	306 (50.9)	154 (50.3)	12.679	<.001	1.806 (1.303‐2.505)	114 (37.3)	6.114	.013	1.542 (1.093‐2.176)
Yes	295 (49.1)	106 (35.9)			Reference	82 (27.8)			Reference
**Smoking status**									
No	550 (91.5)	238 (43.3)	0.000	.985	1.006 (0.563‐1.795)	178 (32.4)	0.182	.669	0.877 (0.481‐1.601)
Yes	51 (8.5)	22 (43.1)			Reference	18 (35.3)			Reference
**Perceived health status**									
Good	536 (89.2)	229 (42.7)	0.583	.445	0.818 (0.488‐1.371)	170 (31.7)	1.810	.179	0.697 (0.411‐1.182)
Bad	65 (10.2)	31 (47.7)			Reference	26 (40)			Reference

**TABLE 3:** Associations between online use behaviors and mental health problems

Variables		Total sample (n, %)	Depression (260; 43.3%)				Anxiety (196; 32.6%)			
			Yes (n, %)	χ^2^ test value	*P*‐value	Odds ratio (95% CI)	Yes (n, %)	χ^2^ test value	*P*‐value	Odds ratio (95% CI)
**Daily internet use time**										
Less than 2 hours		23 (3.8)	6 (26.1)	22.371	<.001	0.323 (0.124‐0.841)	4 (17.4)	19.655	<.001	0.312 (0.104‐0.937**)**
2 to 3 hours		114 (19.0)	38 (33.3)			0.458 (0.293‐0.716)	31 (27.2)			0.553 (0.346‐0.884)
4 to 5 hours		144 (24.0)	49 (34)			0.473 (0.314‐0.711)	32 (22.2)			0.423 (0.269‐0.665)
More than 5 hours		320 (53.2)	167 (52.2)			Reference	129 (40.3)			Reference
**Purpose of online use (yes)**										
Educational	No	95 (15.8)	49 (51.6)	3.180	.075	1.489 (0.960‐2.311)	36 (37.9)	1.433	.231	1.319 (0.837‐2.080)
	Yes	506 (84.2)	211 (41.7)			Reference	160 (31.6)			Reference
Messaging	No	20 (3.3)	5 (25)	2.811	.94	0.426 (0.153‐1.188)	4 (20)	1.497	.221	0.507 (0.167‐1.536)
	Yes	581 (96.7)	255 (43.9)			Reference	192 (33)			Reference
Gaming	No	453 (75.4)	190 (41.9)	1.303	.254	0.805 (0.555‐1.169)	142 (31.3)	1.341	.247	0.795 (0.539‐1.173)
	Yes	148 (24.6)	70 (47.3)			Reference	54 (36.5)			Reference
Video	No	45 (7.5)	17 (37.8)	0.596	.440	0.782 (0.418‐1.462)	14 (31.1)	0.050	.823	0.928 (0.482‐1.788)
	Yes	556 (92.5)	243 (43.7)			Reference	182 (32.7)			Reference
Social media	No	27 (4.5)	10 (37)	0.446	.504	0.762 (0.343‐1.694)	6 (22.2)	1.389	.239	0.577 (0.229‐1.454)
	Yes	574 (95.5)	250 (43.6)			Reference	190 (33.1)			Reference
Shopping	No	473 (78.7)	212 (44.8)	2.199	.138	1.354 (0.906‐2.022)	160 (33.8)	1.490	.222	1.306 (0.850‐2.008)
	Yes	128 (21.3)	48 (37.5)			Reference	36 (28.1)			Reference
News	No	222 (36.9)	108 (48.6)	4.163	**.041**	1.415 (1.013‐1.976)	78 (35.1)	1.020	.313	1.198 (.843 – 1.702)
	Yes	379 (63.1)	152 (40.1)			Reference	118 (31.1)			Reference
Others	No	196 (32.6)	73 (37.2)	4.289	.038	0.692 (0.488‐0.981)	57 (29.1)	1.650	.199	0.785 (0.542‐1.136)
	Yes	405 (67.4)	187 (46.2)			Reference	139 (34.3)			Reference

### 3.2 Prevalence and associated factors for depression and anxiety

The overall prevalence rate of depression and anxiety was 43.3% and 32.6%, respectively. The distribution of depression and anxiety across the variables was presented in Tables 1, 2, and 3. Gender was not significantly associated with depression, whereas slightly higher anxiety was reported in female gender (37% vs 29.4%, *P* = .049). Similarly, higher anxiety level was found among medical college students (39.3% vs 30.5% vs 20.7%, *P* = .042) compared to university and high school students). Considering the relationship status, the students who were engaged in a relationship significantly experienced a higher level of depression (56.7% vs 43.5% vs 25%, *P* = .002) and anxiety (47.8% vs 31.4% vs 25%, *P* = .012) compared to being single or married (Table 1). In addition, individuals sleeping less than 6 hours (50.7%, *P* < .001) were at risk of suffering from higher anxiety levels. Depression and anxiety were strongly significant among those students who were not involved with physical exercise (50.3% vs 35.9%, *P* < .001 and 37.3% vs 27.8%, *P* = .013) (Table 2). Furthermore, using the internet daily for more than 5 hours showed a significant relationship with psychological suffering (52.2%, *P* < .001 for depression and 40.3%, *P* < .001 for anxiety, respectively). Finally, students who were not watching news through online were also depressed (Table 3).

### 3.3 Predictive factors of the students' depression and anxiety

A hierarchical regression analysis was performed with a total of three models for predicting depression and anxiety in Tables 4 and 5. However, sociodemographic information was included in model 1, whereas in model 2, sociodemographic and behavioral health‐related factors and finally in model 3, sociodemographic, behavioral health, and internet use‐related information was included for both depression and anxiety tables. All the models were significantly associated with depression and anxiety except model 1, where *P* = .340 for depression and *P* = .055 for anxiety. Hence, models 2 and 3 predicted only a total of 4.7% and 10% for a variance of depression and 4.7% and 9.6% variance of anxiety, respectively.

**TABLE 4:** Predictive models for the students' depression

Variables	Model 1			Model 2			Model 3		
	[*R* ^2^ = 0.013, *F* = 1.134, adjusted *R* ^2^ = 0.002, *P* = .340]			[*R* ^2^ =0.047, *F* = 2.639, adjusted *R* ^2^ = 0.029, *P* = .003]			[*R* ^2^ = 0.100, *F* = 3.230, adjusted *R* ^2^ = 0.069, *P* < .001]		
	*B*	SE	*β*	*B*	SE	*β*	*B*	SE	*β*
Constant	2.609	0.427		3.431	0.492		1.691	0.696	
Gender ^a^	0.157	0.121	0.055	0.090	0.124	0.031	0.181	0.128	0.063
Educational status ^b^	0.021	0.102	0.008	0.035	0.102	0.014	0.071	0.103	0.029
Current residence ^c^	−0.043	0.140	−0.013	−0.069	0.140	−0.021	−0.081	0.138	−0.025
MFI ^d^	−0.125	0.082	−0.065	−0.136	0.081	−0.071	−0.151	0.080	−0.079
Family type ^e^	−0.084	0.142	−0.024	−0.124	0.141	−0.036	−0.142	0.139	−0.041
Relationship status ^f^	−0.110	0.093	−0.049	−0.141	0.093	−0.062	−0.084	0.093	−0.037
CLWF ^g^	0.188	0.179	0.044	0.116	0.179	0.027	−0.043	0.180	−0.010
DSH ^h^				−0.130	0.092	−0.058	−0.115	0.091	−0.052
Physical exercise ^g^				−0.504	0.120	−0.177	−0.365	0.121	−0.128
Smoking status ^g^				0.210	0.212	0.041	0.222	0.212	0.043
Perceived health status ^i^				0.183	0.187	0.040	0.124	0.184	0.027
DIUT ^j^							0.226	0.066	0.142
Educational ^g^							−0.191	0.166	−0.049
Messaging ^g^							0.875	0.364	0.110
Gaming ^g^							0.197	0.143	0.060
Video watching ^g^							0.018	0.232	0.003
Social media ^g^							0.072	0.297	0.011
Shopping ^g^							−0.103	0.144	−0.030
News ^g^							−0.202	0.126	−0.068
Others ^g^							0.308	0.127	0.101

Abbreviations: CLWF, currently living with the family; DIUT, daily internet use time; DSH daily sleeping hour; MFI, monthly family income.

^j^ 1 = Less than 2 hours, 2 = 2 to 3 hours, 3 = 4 to 5 hours, 4 = More than 5 hours.

^a^ 1 = Male, 2 = Female.

^b^ 1 = University, 2 = Medical college, 3 = High school.

^c^ 1 = Rural, 2 = Urban.

^d^ 1 = Less than 15,000 BDT, 2 = 15,000 to 30,000 BDT, 3 = More than 30,000 BDT.

^e^ 1 = Joint, 2 = Nuclear.

^f^ 1 = Single, 2 = In a relationship, 3 = Married.

^g^ 1 = Yes, 0 = No.

^h^ 1 = Less than 6 hours, 2 = 6 to 7 hours, 3 = More than 7 hours.

^i^ 0 = Good, 1 = Bad.

**TABLE 5:** Predictive models for the students' anxiety

Variables	Model 1			Model 2			Model 3		
	[*R* ^2^ = 0.023, *F* = 1.986, adjusted *R* ^2^ = 0.011, *P* = .055]			[*R* ^2^ = 0.047, *F* = 2.665, adjusted *R* ^2^ = 0.030, *P* = .002]			[*R* ^2^ = 0.096, *F* = 3.083, adjusted *R* ^2^ = 0.065, *P* < .001]		
	*B*	SE	*β*	*B*	SE	*β*	*B*	SE	*β*
Constant	1.443	0.470		2.151	0.544		0.266	0.772	
Gender ^a^	0.278	0.133	0.087	0.250	0.137	0.078	0.360	0.141	0.113
Educational status ^b^	0.151	0.113	0.056	0.173	0.113	0.064	0.198	0.114	0.073
Current residence ^c^	0.267	0.154	0.073	0.273	0.155	0.075	0.261	0.153	0.072
MFI ^d^	−0.143	0.090	−0.067	−0.159	0.090	−0.075	−0.189	0.089	−0.089
Family type ^e^	−0.156	0.157	−0.041	−0.184	0.156	−0.048	−0.199	0.154	−0.052
Relationship status ^f^	−0.032	0.103	−0.013	−0.075	0.103	−0.030	−0.015	0.103	−0.006
CLWF ^g^	0.231	0.197	0.049	0.182	0.198	0.039	−0.022	0.200	−0.005
DSH ^h^				−0.186	0.101	−0.076	−0.176	0.101	−0.072
Physical exercise ^g^				−0.359	0.132	−0.114	−0.216	0.134	−0.069
Smoking status ^g^				0.298	0.234	0.053	0.274	0.235	0.049
Perceived health status^i^				0.392	0.207	0.077	0.337	0.204	0.067
DIUT^j^							0.301	0.073	0.171
Educational ^g^							−0.213	0.184	−0.049
Messaging ^g^							0.763	0.403	0.087
Gaming ^g^							0.313	0.158	0.086
Video watching ^g^							0.108	0.257	0.018
Social media ^g^							0.170	0.329	0.022
Shopping ^g^							−0.093	0.160	−0.24
News ^g^							−0.088	0.140	−0.027
Others ^g^							0.027	0.140	0.008

Abbreviations: CLWF, currently living with the family; DIUT, daily internet use time; DSH, daily sleeping hour; MFI, monthly family income.

^j^ 1 = Less than 2 hours, 2 = 2 to 3 hours, 3 = 4 to 5 hours, 4 = More than 5 hours.

^a^ 1 = Male, 2 = Female.

^b^ 1 = University, 2 = Medical college, 3 = High school.

^c^ 1 = Rural, 2 = Urban.

^d^ 1 = Less than 15,000 BDT, 2 = 15,000 to 30,000 BDT, 3 = More than 30,000 BDT.

^e^ 1 = Joint, 2 = Nuclear.

^f^ 1 = Single, 2 = In a relationship, 3 = Married.

^g^ 1 = Yes, 0 = No.

^h^ 1 = Less than 6 hours, 2 = 6 to 7 hours, 3 = More than 7 hours.

^i^ 0 = Good, 1 = Bad.

## 4 DISCUSSION

The unprecedented COVID‐19 pandemic is a global public health phenomenon keeping people of all ages at an untold threat. Along with impairment related to physical health, it also affects people psychologically more crucially than the disease as a minor portion of the individuals gets infected, whereas people are at risk of psychological uncertainty.^36^ Despite this situation, many studies concerning the mental health effects of the ongoing pandemic were studied in the general Bangladeshi population, though student cohorts have a lack of focus.^8^ In this context, the present study added some findings that may influence policymaking.

In the present study, about 43.3% and 32.6% of the students were reported at risk of suffering from depression and anxiety, respectively; which is lower than that reported for the upper range of mental health problems of the Bangladeshi students (80% and 90%, respectively).^8^ About 34.1% (28.9%–39.4%) and 41.3% (34.7%–48.1%) were the pooled prevalence rates for depression and anxiety, respectively, as reported in a meta‐analysis considering the studies conducted in the South Asian countries.^26^ Besides, Bangladeshi people had been suffering from more psychological problems (i.e., 48.2% [34.8%–61.8%] and 52.3% [41%–63.6%]) as reported in the same review. Again, the present prevalence rates seem to be lower than the pooled rate, where opposite results were reported in a few studies conducted outside Bangladesh.^37^, ^38^ However, the present rates cannot be ignored at all by seeing its surface value because a study assessing a total of 10,056 nationwide participants reported students to be more prone to psychological sufferings (such as depression, suicidal ideation) compared with other cohorts.^39^ In addition, uncertainty related to education may elevate mental health suffering among the students. For instance, lack of concentration on the study, agitation, academic dissatisfaction, and so on influences to increase these problems in the country.^8^ Thus, it is anticipated that the mandatory shutdown of educational institutes for a long time may significantly impact student mental health.

Regarding gender‐based mental health problems distribution, female students reported higher anxiety in the present sample. Although few studies reported there was no significant difference between gender and anxiety, which provided opposite findings of the present study.^25^ Besides, another study reported female gender significantly develops depression more frequently, which is contrast to the present study findings as the present findings reported no association between gender and depression.^40^ Students belonging to medical college were significantly more anxious, which alludes to the stressful academic environment of the cohort leading to suffering from anxiety. In addition, factors such as living in rural areas, had siblings, poor interpersonal relationships, higher academic pressure, poor anticipation of future careers, and sleep deprivation can influence the risk of having higher anxiety.^8^, ^41^ The present study also found that students engaging in a relationship increased the risk of depression and anxiety compared to the single or married one. The finding is not unusual because loneliness and missing out on someone are more common due to the physical distancing and school closure. There is enough evidence on missing partners leading to student suicide during the pandemic.^42^ But, this finding was not explored in any other studies, even in a systematic review that included student cohort during the COVID‐19 pandemic.^8^, ^26^, ^36^ Students with a lack of physical exercise, less sleeping time, and using the internet for a longer duration reported experiencing a higher level of psychological suffering herein. Previous Bangladeshi studies report similar findings during the normal time; for instance, Mamun et al^28^ observed that students using the internet more than 5 hours daily experienced psychiatric problems like depression and anxiety; as consistent with the findings based on the Bangladeshi students mental health related to the pandemic^8^ although internet has also some beneficial foreword. For instance, internet plays an important role in delivering COVID‐19‐related information constantly along with providing psychoeducation, which may have an impact in such a pandemic moment. In addition, online cognitive behavioral therapy can be effective to mitigate mental health issues among the people during the pandemic due to its cost‐effectiveness.

This study provided the predictive models concerning depression and anxiety. The sociodemographic variables predicted only 1.3% and 2.3% of the variance of depression and anxiety, respectively, but the models were not significant. After adjusting behavioral health‐related factors with sociodemographics, 4.7% and 4.7% variance of depression and anxiety, respectively, were explained. However, the final models considering all of the studied variables explained 10% and 9.6% depression and anxiety variance. As being exploratory study in nature, the present models are limited to compare with any other studies.

However, the strength of this study includes observing the relationship between internet use behaviors and psychological outcomes during the COVID‐19 pandemic. Besides, predictive models were reported for the first time among the student's cohort about the variables and the psychological outcomes, facilitating baseline data to this context. The present study also has some limitations, which is worth mentioning. The study was cross‐sectional in nature, which limits the causal inferences, and the sample was nonrepresentative. Besides, selection bias may take place because of an online survey study. In addition, the study was not compared with the pre‐post COVID‐19 situations, which may limit the meaning of the associations found. Furthermore, social desirability or memory recall biases could be placed during the data collection as the data was obtained from a self‐reported online approach. As the study was predominantly used of self‐reported questionnaires to evaluate the psychological outcomes and failed to create clinical diagnosis (e.g., the gold customary for establishing psychological designation concerned structured clinical interview and functional neuroimaging), which might limit the study. Therefore, a further longitudinal study is recommended to recruit a large sample size, which may help understand the situation more precisely.

## 5 CONCLUSIONS

During the COVID‐19 pandemic, the present study found higher psychological suffering among the Bangladeshi students. After being a concerning issue, regrettably, there are no specific policies related to mental health issues in the country. Therefore, the study result may be helpful for the authorities of Bangladesh to take appropriate strategies to assuage these adverse outcomes. The suggested strategies are establishing intervention programs related to mental health awareness, utilizing online media, and providing mental health services through online consultation. Besides, the educational bodies (e.g., high school, university, medical college) should recruit a psychologist who will constantly counsel those students who need urgent psychological care and pay special attention to the risky groups.

## FUNDING

The present study did not get any financial supports. Besides, the authors involved in this research communication do not have any relationships with other people or organizations that could inappropriately influence (bias) the findings.

## CONFLICT OF INTEREST

The authors of the research work do not have any conflict of interest.

## TRANSPARENCY STATEMENT

The authors confirm that the current manuscript is an honest, accurate, and transparent account of the study being reported; that no important aspects of the study have been omitted.

## AUTHOR CONTRIBUTIONS

Conceptualization: Ismail Hosen, Firoj al Mamun, Mohammed A. Mamun

Data Curation: Ismail Hosen, Firoj al Mamun

Formal Analysis: Ismail Hosen, Firoj al Mamun

Investigation: Ismail Hosen, Firoj al Mamun, Mohammed A. Mamun

Methodology: Ismail Hosen, Firoj al Mamun, Mohammed A. Mamun

Resources: Ismail Hosen, Firoj al Mamun, Mohammed A. Mamun

Software: Ismail Hosen, Firoj al Mamun

Supervision: Firoj al Mamun, Mohammed A. Mamun

Validation: Ismail Hosen, Firoj al Mamun, Mohammed A. Mamun

Visualization: Ismail Hosen, Firoj al Mamun, Mohammed A. Mamun

Writing—original draft: Ismail Hosen, Mohammed A. Mamun

Writing—Review and Editing: Ismail Hosen, Firoj al Mamun, Mohammed A. Mamun

All authors have read and approved the final version of the manuscript.

All authors take complete responsibility for the integrity of the data and accuracy of the data.

## Supporting information


**Appendix S1.** Supporting Information

## Data Availability

The data that support the findings of this study are available from the corresponding author upon a reasonable request.
